# Cholesterol metabolism regulation mediated by SREBP-2, LXRα and miR-33a in rainbow trout (Oncorhynchus mykiss) both *in vivo* and *in vitro*

**DOI:** 10.1371/journal.pone.0223813

**Published:** 2020-02-28

**Authors:** Tengfei Zhu, Geneviève Corraze, Elisabeth Plagnes-Juan, Sandrine Skiba-Cassy

**Affiliations:** INRA, Univ Pau & Pays Adour, E2S UPPA, UMR 1419, Nutrition Métabolisme Aquaculture, Saint Pée sur Nivelle, France; National Cheng Kung University, TAIWAN

## Abstract

Cholesterol metabolism is greatly affected in fish fed plant-based diet. The regulation of cholesterol metabolism is mediated by both transcriptional factors such as sterol regulatory element-binding proteins (SREBPs) and liver X receptors (LXRs), and posttranscriptional factors including miRNAs. In mammals, SREBP-2 and LXRα are involved in the transcriptional regulation of cholesterol synthesis and elimination, respectively. In mammals, miR-33a is reported to directly target genes involved in cholesterol catabolism. The present study aims to investigate the regulation of cholesterol metabolism by SREBP-2 and LXRα and miR-33a in rainbow trout using in vivo and in vitro approaches. In vivo, juvenile rainbow trout of ~72 g initial body weight were fed a total plant-based diet (V) or a marine diet (M) containing fishmeal and fish oil. In vitro, primary cell culture hepatocytes were stimulated by graded concentrations of 25-hydroxycholesterol (25-HC). The hepatic expression of cholesterol synthetic genes, *srebp-2* and miR-33a as well as miR-33a level in plasma were increased in fish fed the plant-based diet, reversely, their expression in hepatocytes were inhibited with the increasing 25-HC in vitro. However, *lxrα* was not affected neither in vivo nor in vitro. Our results suggest that SREBP-2 and miR-33a synergistically enhance the expression of cholesterol synthetic genes but do not support the involvement of LXRα in the regulation of cholesterol elimination. As plasma level of miR-33a appears as potential indicator of cholesterol synthetic capacities, this study also highlights circulating miRNAs as promising noninvasive biomarker in aquaculture.

## Introduction

The continuously expanding production of aquaculture since the past decades has posed a great challenge to the supply of fish meal and fish oil which are traditional ingredients in aquafeeds. However, their productions have been keeping stable and will not be promoted in the future due to the quota policy controlling global fisheries captures [1]. Accordingly, vegetable ingredients with lower cost and wider availability have been widely used to replace the fishmeal and fish oil and have achieved considerable advances during the past years [2]. However, the mechanisms underlying the fish physiology affected by vegetable ingredients remain to be investigated. Previous studies have shown that plasma cholesterol level decreased in rainbow trout fed diet with either vegetable oils [3] or plant proteins [4]. This hypocholesterolemia caused by vegetable ingredients was observed in Atlantic salmon [5–7], turbot [8], gilthead sea bream [9–11], and European seabass [12,13]. Expression of genes involved in cholesterol metabolism (cholesterol synthesis, transport, and elimination) were found to be affected by vegetable ingredients in Atlantic Salmon and European seabass [7,14–16]. As cholesterol is not only an essential component of the membranes [17] but also the precursors of several bioactive compounds, including bile acids [18], steroid hormones [19] and vitamin D [20], the alteration of cholesterol metabolism could inevitably result in an array of consequences that may affect the normal physiology of the fish.

The processes and pathways of cholesterol metabolism are quite similar in fish and mammals. Dietary cholesterol is incorporated with bile acids in micelles and then absorbed by enterocytes or pyloric caeca in fish [21,22]. Fish are also able to synthesize cholesterol per se, mainly in the liver with a metabolic process including more than 20 reactions [23]. Numerous enzymes, receptor proteins and different kinds of lipoproteins are involved in the transport of cholesterol to peripheral tissues via circulating system [24]. In addition to the direct cholesterol excretion by the liver and the intestine, the bile acid synthesis in liver also contributes to cholesterol elimination in fish, which is similar to mammals as well [25].

Cholesterol metabolism in mammals is known to be tightly regulated at transcriptional level by sterol regulatory element-binding proteins (SREBPs) and liver X receptors (LXRs). The SREBP family includes three isoforms: SREBP-1a, -1c and -2. Among them, SREBP-1a is able to activate all SREBP-responsive genes including those involved in the syntheses of cholesterol, fatty acids, and triglycerides, while SREBP-1c and -2 preferentially activate genes related to fatty acid and cholesterol synthesis, respectively [26,27]. There are two isoforms of LXRs in mammals, termed LXRα and β, which are nuclear receptors serving as lipid sensors in case of lipid overload [28]. LXRα is predominantly expressed in liver, intestine, and adipose tissue, while LXRβ is expressed at lower level but ubiquitously [29,30]. The genes involved in cholesterol elimination have been reported to be directly activated by LXRs [31,32].

MicroRNAs (miRNAs) are a class of small non-coding RNAs with about 22 nucleotides in length and serve for posttranscriptional regulation of gene expression by binding with 3’ untranslated region (UTR) of the target genes, resulting in mRNA cleavage or transcriptional repression [33–35]. A miRNA can regulate more than 200 genes and each mRNA in turn has multiple binding sites of miRNAs, constructing an extensive and complicated network for fine regulation of gene expression [36,37].The high degree of sequence conservation across distantly related species further suggests essential role of miRNAs in biological process. As expected, many fundamental biological processes have been reported to be regulated by miRNAs, including animal development [38], cell differentiation [39], signal transduction [40], metabolism [41], disease [42] and apoptosis [43]. Likewise, numbers of genes involved in various processes of cholesterol metabolism are reported to harbor target sites of miRNAs in their 3’UTR and are regulated by miRNAs. Among them, many studies in mammals have investigated the role of miR-33 in cholesterol metabolism. In human, two isoforms of miR-33 were identified, miR-33a located in the intron 16 of SREBP-2 and miR-33b, located in the intron 17 of SREBP-1, whereas, only miR-33a is present in mice [44]. Studies in mammals showed that miR-33a was co-expressed with SREBP-2 [45–47] and target sites of miR-33a were located in the 3’UTR of cholesterol efflux related genes such as transporter ATP-binding cassette A1 (ABCA1) and ABCG1 [45], bile acid synthesis enzyme cholesterol 7α-hydroxylase (CYP7A1) [47], and biliary secretion transporter like ABCB11 [46]. Some of these studies also indicated that these genes were inhibited by miR-33(a) at mRNA or protein level. Plasma level of high-density lipoproteins (HDL) and biliary secretion were correspondingly decreased by miR-33(a) as well [44–48]. Though the pivotal roles of miRNAs in the development and metabolism of fish have been investigated in some studies (for review see [49], few of them reported their involvement in cholesterol metabolism of fish fed plant-based diet.

Therefore, in the present study, we performed an in vivo experiment with trout fed either plant-based diet or marine diet and an in vitro assay based on stimulation of primary cell culture of rainbow trout hepatocyte with graded levels of 25-hydroxycholesterol to evaluate the involvement of SREBP-2, LXRα and miR-33a in the mechanisms underlying the alteration of cholesterol metabolism in rainbow trout.

## Materials and methods

### 1. Ethics statement

Experiments were carried out in the INRA experimental facilities (UMR1419 Nutrition, Métabolisme, Aquaculture, Donzacq, France) authorized for animal experimentation by the French veterinary service which is the competent authority (A 64-495-1). Experiments were in strict accordance with EU legal frameworks related to the protection of animals used for scientific research (Directive 2010/63/EU) and according to the National Guidelines for Animal Care of the French Ministry of Research (decree n°2013–118, February 1st, 2013). Scientists in charge of the experimentation received a training and a personal authorization (N°B64 10 005). In agreement with the ethical committee “Comité d’Ethique Aquitaine Poissons Oiseaux” (C2EA-73), the present study does not need approval by a specific ethical committee since it implies only classical rearing practices with all diets formulated to cover the nutritional requirements of rainbow trout. During this study, fish were daily monitored. If any clinical symptoms (i.e. morphological abnormality, restlessness or uncoordinated movements) were observed, fish were sedated by immersion in 10mg/L benzocaine solution and then euthanized by immersion in a 60mg/L benzocaine solution (anesthetic overdose) during 3 minutes.

### 2. Experimental design

In order to investigate how cholesterol metabolism is affected at transcriptional and post-transcriptional levels in rainbow trout by the transcription factors SREBP-2 and LXRα a and the miR-33a, respectively, we designed both in vivo and in vitro experiments. For the in vivo experiment, the fish were fed either a totally plant-based diet (V) or the marine diet (M) containing fishmeal and fish oil for 10 weeks. In vitro, primary cell culture of rainbow trout hepatocyte were prepared and stimulated by graded levels of 25-hydroxycholesterol which served as a reflection of short-term elevation of cellular cholesterol level. Finally, the genes related to cholesterol-metabolism and miR-33a were investigated in both experiments in order to decipher the regulation of cholesterol and lipid metabolism.

### 3. Diets, fish and sampling procedure

Two diets formulated to be isonitrogenous and isolipidic were manufactured in our experimental facilities of Donzacq (France) with a twin-screw extruder (Clextral, Firminy, France). Diet M contained fishmeal and fish oil as protein and lipid source, respectively. Diet V contained a blend of vegetable oils (palm, rapeseed and linseed oils) and a blend of plant protein sources. Diets were formulated to fulfill the requirements of rainbow trout according to NRC recommendations [50]. Synthetic L-lysine, L-arginine, dicalcium-phosphate and soy-lecithin were added to diet V to meet the requirement of essential amino acids, phosphorous and phospholipid in rainbow trout. Diet formulation and composition are showed in **Table 1**.

**Table 1:** Ingredients and analytical composition of the diets.

Ingredients (%)	V		M
Fish meal	0.00	58.42
Corn gluten	16.03	0.00
Wheat gluten	17.72	0.00
Soybean meal	10.13	0.00
Soy protein concentrate	15.19	0.00
Light white lupin	6.75	0.00
Dehulled pea	4.30	0.00
Extruded whole wheat,	3.38	25.26
Fish oil	0.00	14.09
Vegetable oils^1^	15.61	0.00
Mineral premix^2^	1.18	1.12
Vitamin premix^3^	1.18	1.12
Soy lecithin	2.11	0.00
L-lysine	1.52	0.00
L-methionine	0.34	0.00
CaHPO4.2H2O	3.21	0.00
Attractant Mix	1.35	0.00
*Analytical composition*		
Dry Matter (DM, %)	94.41	90.13
Crude protein (% DM)	50.01	46.98
Lipid (% DM)	19.49	18.80
Sterols (% DM)	0.004	0.737
Energy (kJ/g DM)	23.84	23.24
Ash (% DM)	6.25	8.17

^1^ Vegetable oils: palm oil (30%), rapeseed oil (55%), linseed oil (15%)

^2^ Mineral premix (g or mg/kg diet): calcium carbonate (40% Ca), 2.15 g; magnesium oxide (60% Mg), 1.24 g; ferric citrate, 0.2 g; potassium iodide (75% I), 0.4 mg; zinc sulfate (36% Zn), 0.4 g; copper sulfate (25% Cu), 0.3 g; manganese sulfate (33% Mn), 0.3 g; dibasic calcium phosphate (20% Ca, 18% P), 5 g; cobalt sulfate, 2 mg; sodium selenite (30% Se), 3 mg; KCl, 0.9 g; and NaCl, 0.4 g (UPAE, INRA).

^3^ Vitamin premix (IU or mg/kg diet): DL-α-tocopherol acetate, 60 IU; sodium menadione bisulphate, 5 mg; retinyl acetate, 15,000 IU; DL-cholecalciferol, 3000 IU; thiamin, 15 mg; riboflavin, 30 mg; pyridoxine, 15 mg; B12, 0.05 mg; nicotinic acid, 175 mg; folic acid, 500 mg; inositol, 1000 mg; biotin,2.5 mg; calcium panthotenate,50mg; and choline chloride, 2000 mg (UPAE, INRA).

Rainbow trout with approximately 72 g initial body weight were reared in our experimental fish farm (INRA, Donzacq, France-permit n°A64-104-1) in open circuit tanks supplied with spring water at 17°C and under natural photoperiod. Fish were randomly distributed into six tanks (70–80 fish per 300-liter tank; three tanks per treatment) and fed twice a day until apparent satiation with either the plant-based diet (V) or the marine diet (M) for 10 weeks.

At the end of the experiment, nine fish were sampled from each tank 8 h after the last meal, anesthetized with benzocaine (30 mg/L) and killed by a sharp blow to the head. Blood was removed from the caudal vein into syringes rinsed with 10% EDTA and centrifuged (3000 g, 5min). The recovered plasma was immediately frozen and kept at -80°C for miRNA expression (six fish) and plasmatic parameters (nine fish) analyses. Liver and viscera from nine fish were dissected and weighed for viscerosomatic and hepatosomatic index determination, respectively. Liver was then immediately frozen in liquid nitrogen and kept at -80°C for gene and miRNA expression analysis.

### 4. Diet and whole body composition analysis

Proximate analysis of the experimental diets and whole body was determined according to the Association of Official Analytical Chemists [51] as follows: Dry matter was analyzed by drying the samples to constant weight at 105°C for 24 h. Crude protein was determined using the Kjeldahl method after acid digestion and estimated by multiplying nitrogen by 6.25. Crude lipid was quantified by petroleum diethyl ether extraction using the Soxhlet method. Gross energy content was determined in an adiabatic bomb calorimeter (IKA). Ash was examined by combustion in a muffle furnace at 550°C for 16 h.

### 5. Plasma metabolites analysis

Plasma glucose, triglycerides and cholesterol concentrations were measured on nine fish per diet using commercial kits (Sobioda, France) adapted to microplate format, according to the recommendations of the manufacturers.

### 6. Hepatocyte cell culture

#### 6.1 Animals

Rainbow trout were maintained in tanks of open circuits with 18°C and well-aerated water in INRA experimental fish facilities of Saint Pée sur Nivelle, France and fed a commercial diet (T-3P classic, Trouw, France). After two days fasting, trout were chosen for hepatocyte isolation.

#### 6.2 Hepatocyte isolation and culture

Isolated liver cells were prepared as previously described [52]. Firstly, fish were anesthetized in a bath containing 30 mg L^–1^ benzocaine and then killed using a 60 mg·L^−1^ benzocaine bath. Livers excised and minced with a razor blade after in situ perfusion with liver perfusion medium (1×, 17701–038, Invitrogen, Carlsbad, CA, USA). The minced livers were then immediately digested in liver digest medium at 18°C for 20 min. After filtration and centrifugation (120 g, 2min), the resulting cell pellet was resuspended and centrifuged (70 g, 2min) three times successively in modified Hanks’ medium (136.9 mmol L^–1^ NaCl, 5.4 mmol L^–1^ KCl, 0.81 mmol L^–1^ MgSO_4_, 0.44 mmol L^–1^ KH_2_PO_4_, 0.33 mmol L^–1^ Na_2_HPO_4_, 5 mmol L^–1^ NaHCO_3_ and 10 mmol L^–1^ Hepes) supplemented with 1.5 mmol L^–1^ CaCl_2_ and 1.5% defatted bovine serum albumin (BSA; Sigma Aldrich, Saint Quentin Fallavier, France). Cells were finally taken up in modified Hanks’ medium supplemented with 1.5 mmol L^–1^ CaCl_2_, 1% defatted BSA, 3 mmol L^–1^ glucose, MEM essential amino acids (1×, Invitrogen, Carlsbad, CA, USA), MEM non-essential amino acids (1×, Invitrogen, Carlsbad, CA, USA) and antibiotic antimycotic solution (1×, Sigma).

Cell viability (>98%) was assessed using the Trypan Blue exclusion method (0.04% in 0.15 mol L^–1^ NaCl) and cells were counted with a hemocytometer. The hepatocyte cell suspension (CS) was plated in six-well Primaria culture dish (BD Biosciences, NJ, USA) at a density of 3×10^6^ cells per well and incubated at 18°C. The incubation medium was replaced every 24 h over the 48 h of primary cell culture. Microscopic examination ensured that hepatocytes progressively re-associated throughout culture to form two-dimensional aggregates, in agreement with earlier reports [53,54].

#### 6.3 Primary hepatocyte stimulated by graded levels of hydroxycholesterol

The 48h-cultured hepatocytes were stimulated with five graded levels (0, 1, 2, 3, 4 mg L-1) of 25-hydroxycholesterol (25-HC) (H1015, Sigma Aldrich, Saint Quentin Fallavier, France) and designated as C0, C1, C2, C3, C4, respectively, using ethanol as the solvent. After 16 h culturing with 25-HC, the hepatocytes were harvested in TRIzol Reagent (Invitrogen, Carlsbad, CA, USA) for mRNA extraction and subsequent gene and miRNA expression analysis. The cell culture experiment was repeated twice for confirmation.

### 7. Gene expression analysis in liver and hepatocytes

Quantitative RT-PCR gene expression analyses were performed on liver (n = 6) and hepatocytes (n = 3). Genes studied were ATP-binding cassette transporter A1 (*abca1*), ATP-binding cassette transporter G5 (*abcg5*), ATP-binding cassette transporter G8 (*abcg8*), cholesterol 7α-hydroxylase (*cyp7a1*), HMG-CoA reductase (*hmgcr*), HMG-CoA synthase (*hmgcs*), sterol regulatory element-binding protein 2 (*srebp-2*), liver X receptor α (*lxrα*), 7-dehydrocholesterol reductase (*dhcr7*), UDP glycuronosyltransferase (*ugt1a3*), Lanosterol 14α-demethylase (*cyp51*), fatty acid synthase (*fas*), sterol regulatory element-binding protein 1c (*srebp-1c*), and glucokinase (*gck*). Total RNA was extracted as previously described [55] using the Trizol reagent (Invitrogen, Carlsbad, CA, USA) according to the manufacturer's instructions and was quantified by spectrophotometry (absorbance at 260nm). The integrity of the samples was assessed using agarose gel electrophoresis. 1 μg of total RNA was used for cDNA synthesis. The SuperScript III RNaseH-reverse transcriptase kit (Invitrogen) with oligo dT random primers (Promega, Charbonniéres, France) was used to synthesize cDNA (n = 6 for cholesterol metabolism genes in 6h). The primer sequences used for qRT-PCR analyses are listed in **Table 2**. Quantitative RT-PCR assays were performed on the Roche LightCycler 480 II system (Roche Diagnostics, Neuilly sur Seine, France). The assays were carried out using a reaction mix of 6 μL per sample containing 2 μL of 76 times diluted cDNA, 0.24 μL of each primer (10 μM), 3 μL of LightCycler 480 SYBR^®^ Green I Master mix (ThermoFisher Scientific, Waltham, USA) and 0.52 μL DNAse/RNAse free water (5 Prime GmbH, Hamburg, Germany). The PCR protocol was initiated at 95°C for 10 min for initial denaturation of the cDNA and hot-start Taq-polymerase activation, followed by 45 cycles of a three-step amplification program (15s at 95°C, 10s at melting temperature Tm (60–65°C), 15s at 72°C), according to the primer set used. Melting curves were systematically monitored (5 s at 95°C, 1 min at 65°C, temperature gradient at 0.11°C/s from 65 to 97°C) at the end of the last amplification cycle to confirm the specificity of the amplification reaction. Each PCR assay included replicate samples (duplicate of reverse transcription and PCR amplification) and negative controls (RT- and cDNA-free samples, respectively). Elongation factor 1α (*ef1α*) showed no significant difference among treatments and was used for the gene normalization. Relative quantification of target gene expression was determined using the E-Method from the LightCycler 480 software (version SW 1.5; Roche Diagnostics). In all cases, PCR efficiency (E) measured by the slope of a standard curve with serial dilutions of cDNA ranged between 1.8 and 2.

**Table 2:** Sequences of the primer pairs used for gene expression analysis by qRT-PCR.

			Genoscope^1^ or Genbank accession numbers	
*Gene*	Forward primer	Reverse primer	Paralogue 1	Paralogue 2
*abca1*	CAGGAAAGACGAGCACCTT	TCTGCCACCTCACACACTTC	GSONMG00078741001	GSONMG00074045001
*abcg5*	CACCGACATGGAGACAGAAA	GACAGATGGAAGGGGATGAA	GSONMG00075025001	/
*abcg8*	GATACCAGGGTTCCAGAGCA	CCAGAAACAGAGGGACCAGA	GSONMG00075024001	/
*cyp51*	CCCGTTGTCAGCTTTACCA	GCATTGAGATCTTCGTTCTTGC	GSONMG00031182001	GSONMG00044416001
*cyp7a1*	ACGTCCGAGTGGCTAAAGAG	GGTCAAAGTGGAGCATCTGG	AB675933.1 GSONMG00066448001	AB675934.1 GSONMG00037174001
*dhcr7*	GTAACCCACCAGACCCAAGA	CCTCTCCTATGCAGCCAAC	GSONMG00025402001	GSONMG00039624001
*fas*	TGATCTGAAGGCCCGTGTCA	GGGTGACGTTGCCGTGGTAT	GSONMG00062364001	/
*gck*	GCACGGCTGAGATGCTCTTTG	GCCTTGAACCCTTTGGTCCAG	GSONMG00033781001	GSONMG00012878001
*hmgcr*	GACCATTTGGGAGCTTGTGT	GAACGGTGAATGTGCTGTGT	GSONMG00016350001	/
*hmgcs*	AGTGGCAAAGAGAGGGTGTG	TTCTGGTTGGAGACGAGGAG	GSONMG00010243001	/
*lxrα*	TGCAGCAGCCGTATGTGGA	GCGGCGGGAGCTTCTTGTC	GSONMG00014026001	GSONMG00064070001
*srebp-1c*	CATGCGCAGGTTGTTTCTT	GATGTGTTCGTGTGGGACTG	XM_021624594.1	/
*srebp-2*	TAGGCCCCAAAGGGATAAAG	TCAGACACGACGAGCACAA	GSONMG00039651001	GSONMG00061885001
*ugt1a3*	CCACCAGCAAGACAGTCTCA	CAACAGCACAGTGGCTGACT	GSONMG00035844001	/
*ef1α*	TCCTCTTGGTCGTTTCGCTG	ACCCGAGGGACATCCTGTG	AF498320.1	/

^1^ https://www.genoscope.cns.fr/trout/

### 8. miRNA expression analysis in liver, hepatocytes and plasma

miR-33a-5p (478347_mir GTGCATTGTAGTTGCATTGCA, ThermoFisher Scientific, Waltham, USA) was detected by the TaqMan^®^ Advanced miRNA Assays (A25576, ThermoFisher Scientific, Waltham, USA) in liver. The spike miR-39-3p (*C*. *elegans*) [56] (478293_mir TCACCGGGTGTAAATCAGCTTG, ThermoFisher Scientific, Waltham, USA) was used for miR-33a normalization and it was present at relatively constant levels among the treatments. Total RNA in liver was obtained in the same way as that for gene expression analysis. 80ng RNA were used for the poly(A) tailing, ligation and reverse transcription reactions to synthesize the cDNA of all miRNAs followed by a miR-Amp reaction for cDNA pre-amplification according to the manufacturer’s instruction. PCR was performed in a reaction mix of 6 μL containing 2 μL cDNA (200 times diluted for liver cDNA and 50 times diluted for plasma cDNA), 2.67 μL 2X Fast Advanced Master mix (ThermoFisher Scientific, Waltham, USA), 0.27 μL TaqMan^®^ Advanced miRNA Assay (20X) (ThermoFisher Scientific, Waltham, USA) and 1.06 μL DNAse/RNAse free water (5 Prime GmbH, Hamburg, Germany). The PCR protocol was initiated at 95°C for 20s for initial denaturation of the cDNA and the enzyme activation, followed by 50 cycles of a 2 steps amplification program (3s at 95°C for denaturation, 30s at 60°C for annealing). Each PCR assay included replicates for each sample (duplicates of reverse transcription and PCR amplification) and negative controls (reverse transcriptase free and RNA free samples). Relative quantification of the target miRNA was determined using the E-Method from the LightCycler 480 software (version SW 1.5; Roche Diagnostics, Meylan, France). PCR efficiency measured by the slope of a standard curve with serial dilutions of miRNA cDNA ranged between 1.8 and 2.

Correspondingly, miR-33a-5p was also detected in plasma samples by the TaqMan^®^ Advanced miRNA Assays (A25576, ThermoFisher Scientific, Waltham, USA). miR-39-3p was added and used as an exogenous control for normalization and showed constant levels in plasma samples. Total RNA was extracted from plasma samples with the TRIzol LS reagent (Life Technologies, Carlsbad, CA, USA) according to the manufacturer's instructions and was quantified by spectrophotometry (absorbance at 260nm). The cDNA synthesis of all miRNAs and the following qPCR steps were performed in the same way as those previously described for hepatic miRNA expression analysis. PCR efficiency measured by the slope of a standard curve with serial dilutions of miRNA cDNA was nearly 2.0.

### 9. Statistical analysis

Results are expressed as means ± SD (n = 3 for body composition and gene and miRNA expression in vitro; n = 6 for gene and miRNA expression in vivo; n = 9 for hepatosomatic, viscerosomatic index and plasma parameters). Statistical analyses were carried out using one-way ANOVA, followed by a Tukey test for post hoc analysis. Normality was beforehand assessed using the Shapiro-Wilk test, while homogeneity of variance was determined using Levene’s test. For all statistical analysis, the level of significance was set at *P*<0.05. Pearson correlation coefficients were calculated based on data of normalized genes or miRNA expression calculated by the E-Method from the LightCycler 480 software (version SW 1.5; Roche Diagnostics, Meylan, Fance). Statistical analyses were performed using R software [57].

## Results

### 1. The effect of plant-based diet on growth, body composition and plasma parameters in vivo experiment

After a 10-week trial, the final body weight (FBW) was significantly decreased in trout fed plant-based diet despite the slightly higher level of protein in the plant-based diet. The hepatosomatic index (HSI) and viscerosomatic index (VSI) of trout were not affected by the diets as fish body composition, including protein, lipid, ash and energy contents. Regarding plasma parameters, with exception of cholesterol which was significantly decreased in trout fed plant-based diet, neither triglycerides nor glucose was affected by the diets. (**Table 3**)

**Table 3:** Growth performance, body composition and plasma parameters of rainbow trout fed the experimental diets for ten weeks.

	M		V		
	Mean	SD	Mean	SD	*P* value
Final body weight (g)	311.44^a^	40.87	223.72^b^	23.91	<0.001
HSI (%)	1.34	0.28	1.15	0.12	0.337
VSI (%)	13.14	1.14	13.30	0.68	1.000
*Body composition*					
Dry matter (DM, %)	31.57	1.04	31.70	0.38	0.845
Protein (% DM)	51.69	0.67	51.97	0.31	0.536
Lipid (% DM)	40.20	1.68	40.30	0.47	0.923
Ash (% DM)	6.22	0.48	6.65	0.15	0.222
Energy (Kg/g DM)	28.56	0.51	28.61	0.16	0.863
*Plasma parameters*					
Cholesterol (g L^-1^)	3.55^a^	0.85	2.10^b^	0.25	<0.001
Triglyceride (g L^-1^)	3.46	2.31	3.87	1.41	0.651
Glucose (g L^-1^)	0.85	0.20	0.87	0.11	0.809

^a, b^ Mean values with different superscript letters were significantly different (*P*<0.05; One-way analysis of variance, Tukey’s test)

HSI, Hepatosomatic index = 100 × liver weight/body weight; VSI, Viscerosomatic index = 100 × viscera weight/body weight.

### 2. Expression of genes involved in cholesterol metabolism

#### 2.1 In vivo experiment

**Fig 1:**
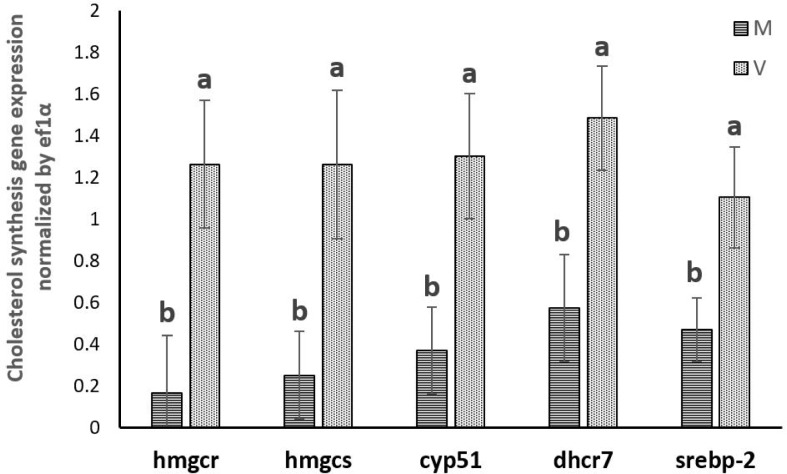
Gene expression involved in cholesterol synthesis in the liver of rainbow trout fed marine (M) diet and (V) vegetable diet. Expression values were normalized by ef1α. Values are means (n = 6), with their standard deviations represented by vertical bars. ^a, b^ Mean values with unlike letters were significantly different (*P*<0.05, One-way analysis of variance, Tukey’s Test).

**Fig 2:**
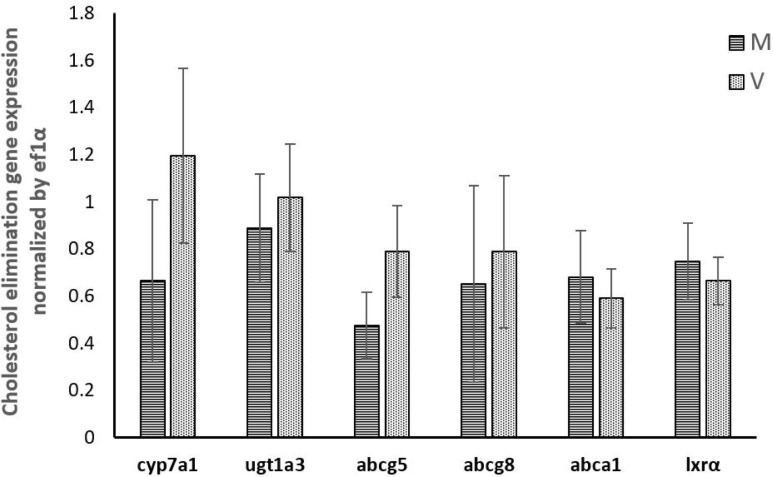
Gene expression involved in cholesterol elimination in the liver of rainbow trout fed marine (M) diet and (V) vegetable diet. Expression values were normalized by ef1α. Values are means (n = 6), with their standard deviations represented by vertical bars. (*P*>0.05, One-way analysis of variance, Tukey’s Test).

**Fig 3:**
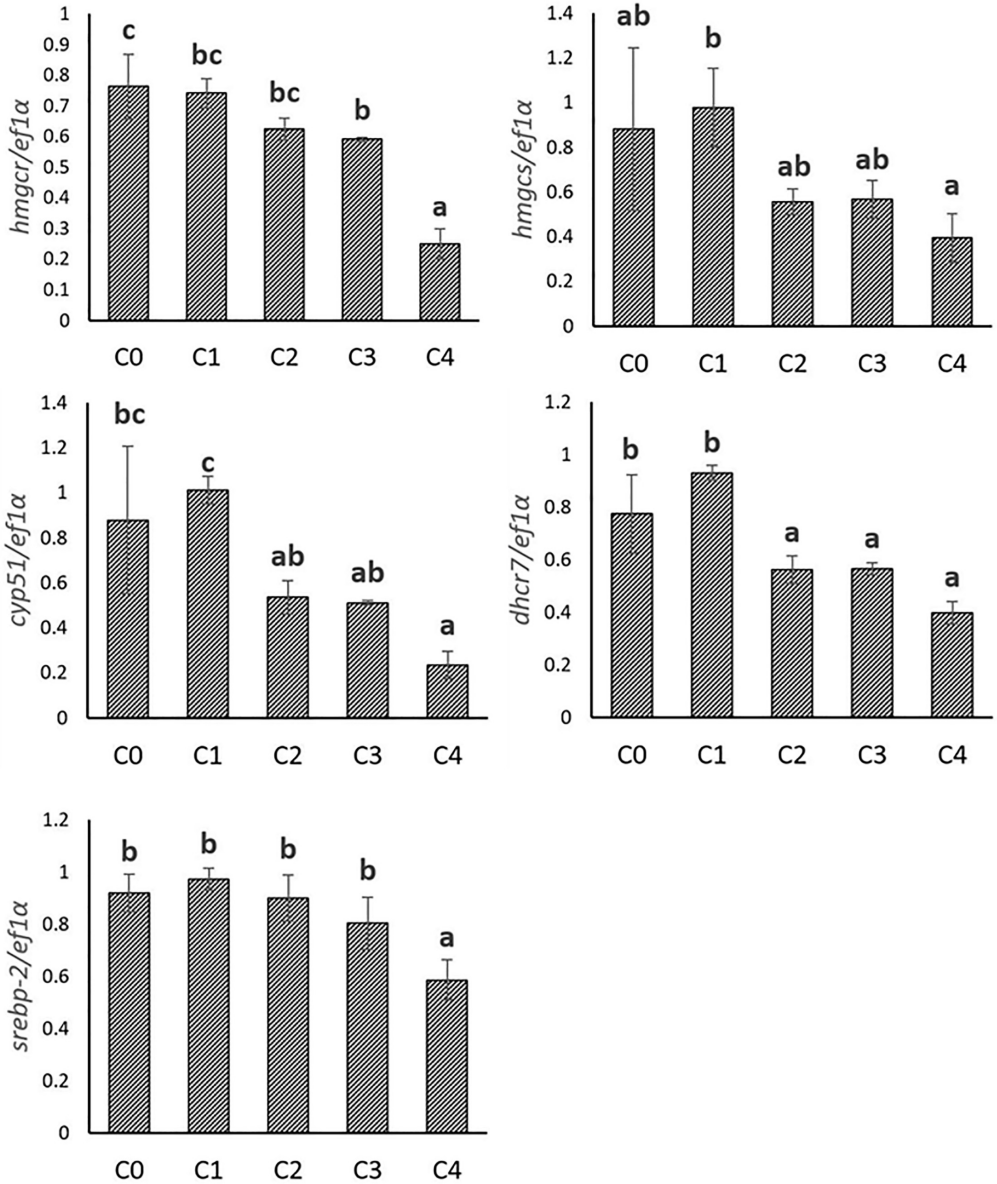
Gene expression involved in cholesterol synthesis in trout hepatocytes stimulated with graded levels of 25-hydroxycholesterol (25-HC) in cell culture. Five graded concentrations, 0, 1, 2, 3, 4 mg/L 25-HC are designated as C0, C1, C2, C3, C4, respectively. Expression values were normalized by ef1α. Values are means (n = 3), with their standard deviations represented by vertical bars. ^a, b, c^ Mean values with unlike letters were significantly different (*P*<0.05, One-way analysis of variance, Tukey’s Test).

The expression of genes involved in cholesterol synthesis (*hmgcr*, *hmgcs*, *cyp51*, *dhcr7* and *srebp-2*) determined in the present study was significantly promoted in trout fed plant-based diet. On the contrary, none of the genes involved in cholesterol elimination (*cyp7a1*, *ugt1a3*, *abcg5*, *abcg8*, *abca1* and *lxrα*) showed significantly different expressions between trout fed marine diet or plant-based diet. (**Figs 1–3**)

#### 2.2 In vitro experiment

**Fig 4:**
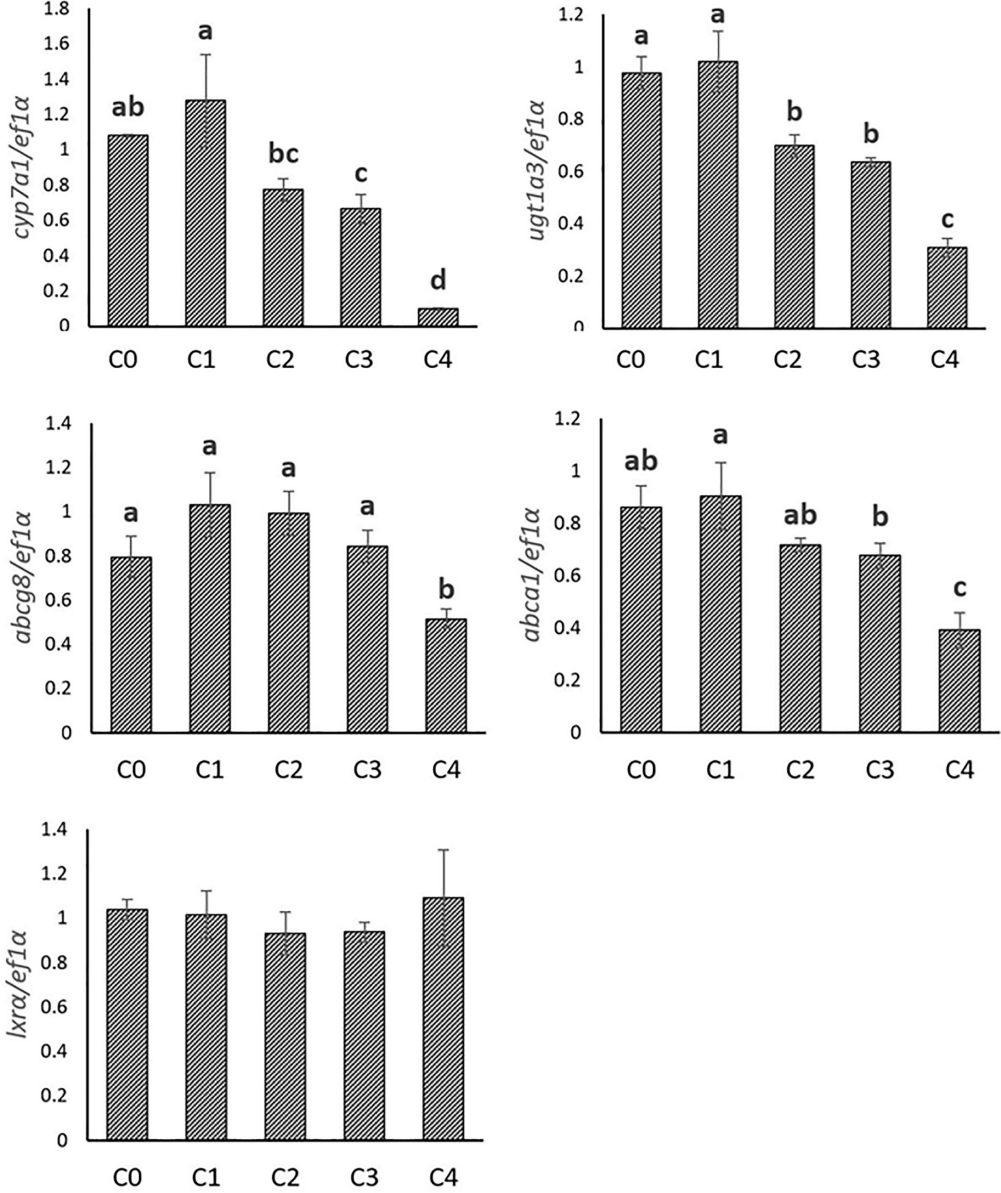
Gene expression involved in cholesterol elimination in trout hepatocytes stimulated with graded levels of 25-hydroxycholesterol (25-HC) in cell culture. Five graded concentrations, 0, 1, 2, 3, 4 mg/L 25-HC are designated as C0, C1, C2, C3, C4, respectively. Expression values were normalized by ef1α. Values are means (n = 3), with their standard deviations represented by vertical bars. ^a, b, c, d^ Mean values with unlike letters were significantly different (*P*<0.05, One-way analysis of variance, Tukey’s Test).

The expression of genes involved in cholesterol metabolism was also evaluated in the hepatocytes stimulated by 25-HC. The expression of all cholesterol synthetic genes (*hmgcr*, *hmgcs*, *cyp51*, *dhcr7* and *srebp-2*) was significantly inhibited with the increasing levels of 25-HC. Similarly, the expression of some genes involved in cholesterol elimination (*cyp7a1*, *ugt1a3*, *abcg8*, and *abca1*) was also found to be significantly decreased when 25-HC concentration increased. By contrast, *lxrα* was not affected by increasing levels of 25-HC. (**Figs 3 and 4**)

### 3. Expression of genes involved in lipogenesis

**Fig 5:**
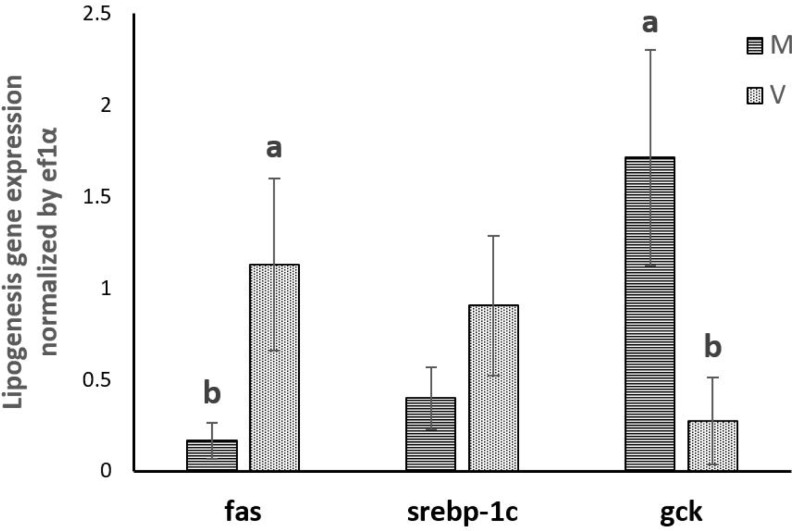
Gene expression involved in lipogenesis in the liver of rainbow trout fed marine (M) diet and (V) vegetable diet. Expression values were normalized by ef1α. Values are means (n = 6), with their standard deviations represented by vertical bars. ^a, b^ Mean values with unlike letters were significantly different (*P*<0.05, One-way analysis of variance, Tukey’s Test).

In vivo, the expression of *fas* was significantly increased in trout fed plant-based diet, whereas *srebp-1c* expression remained stable. The expression of *gck* was significantly decreased in trout fed plant-based diet. (**Fig 5**)

**Fig 6:**
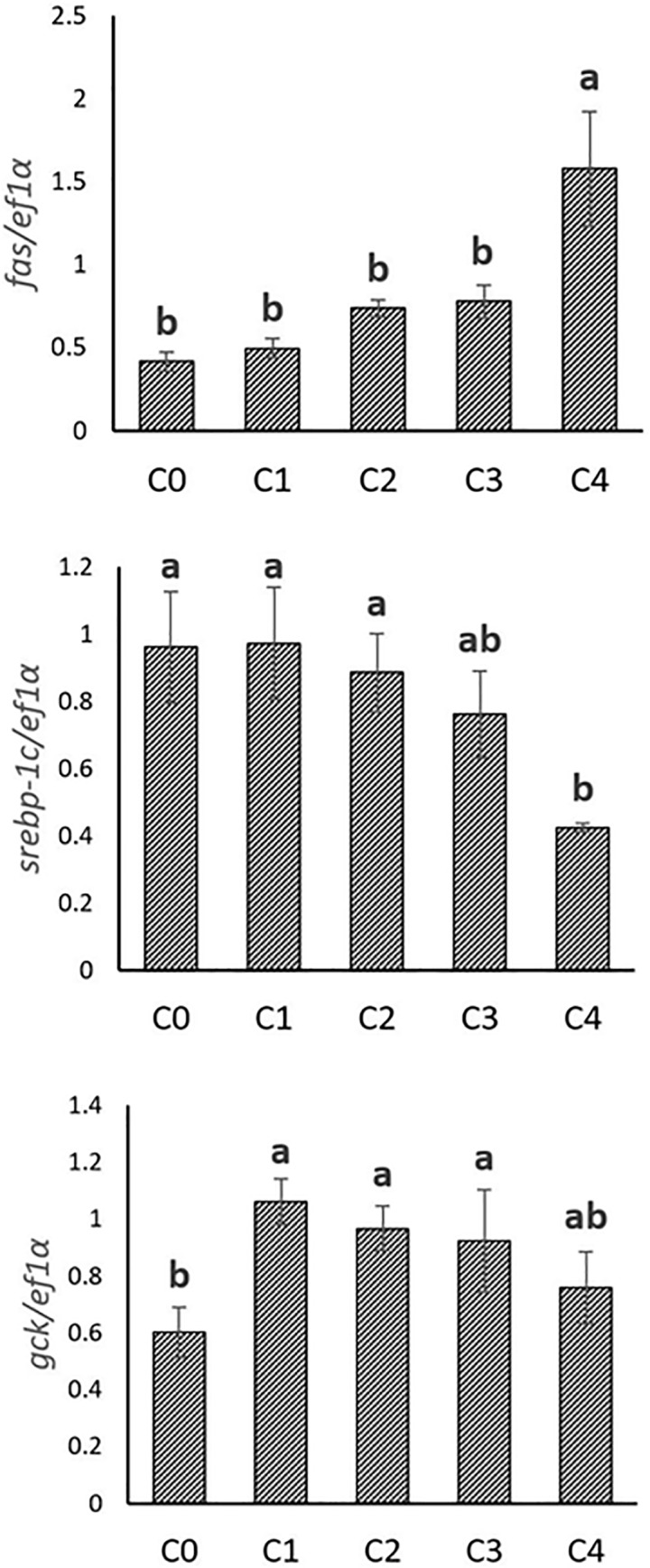
Gene expression involved in lipogenesis in trout hepatocytes stimulated with graded levels of 25-hydroxycholesterol (25-HC) in cell culture. Five graded concentrations, 0, 1, 2, 3, 4 mg/L 25-HC are designated as C0, C1, C2, C3, C4, respectively. Expression values were normalized by ef1α. Values are means (n = 3), with their standard deviations represented by vertical bars. ^a, b^ Mean values with unlike letters were significantly different (*P*<0.05, One-way analysis of variance, Tukey’s Test).

In vitro, *fas* was more expressed in hepatocytes when the level of 25-HC increased, while the expression of *srebp-1c* was significantly inhibited with increasing levels of 25-HC. The expression of *gck* was significantly increased by 25-HC at the concentration from C1 to C3. At the highest concentration (C4), the level of expression of gck was no more different from all the other treatments. (**Fig 6**)

### 4. miR-33a expression and plasma abundance

**Fig 7:**
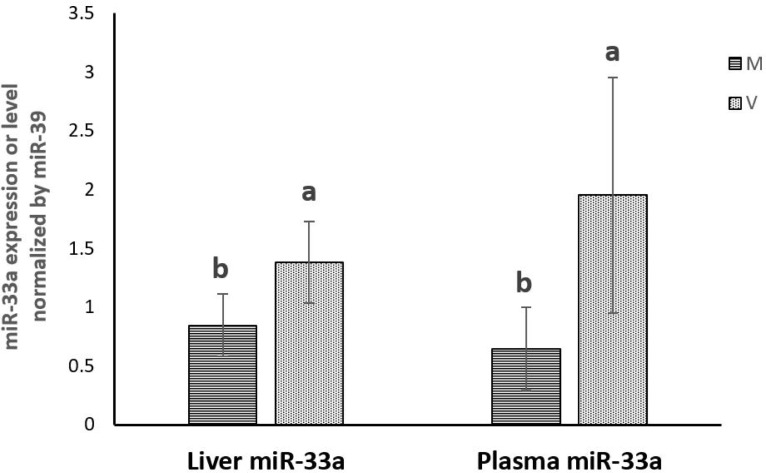
miR-33a expression in the liver and level in the plasma of rainbow trout fed marine (M) diet and (V) vegetable diet. Expression values were normalized by miR-39. Values are means (n = 6), with their standard deviations represented by vertical bars. ^a, b^ Mean values with unlike letters were significantly different (*P*<0.05, One-way analysis of variance, Tukey’s Test).

**Fig 8:**
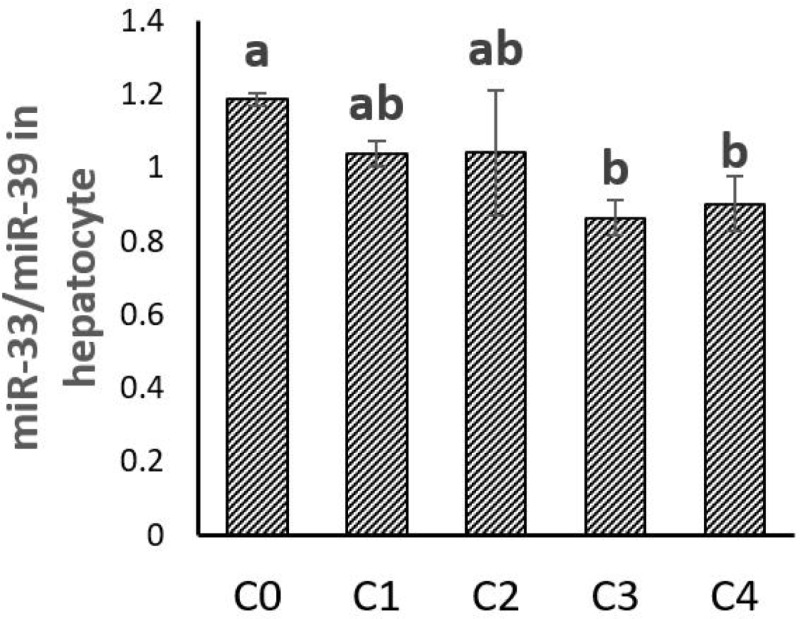
miR-33a expression in the hepatocytes stimulated with graded levels of 25-hydroxycholesterol (25-HC) in cell culture. Five graded concentrations, 0, 1, 2, 3, 4 mg/L 25-HC are designated as C0, C1, C2, C3, C4, respectively. Expression values were normalized by miR-39. Values are means (n = 3), with their standard deviations represented by vertical bars. ^a, b^ Mean values with unlike letters were significantly different (*P*<0.05, One-way analysis of variance, Tukey’s Test).

The hepatic expression and the plasma abundance of miR-33a were both significantly increased in trout fed plant-based diet in vivo. Results of the in vitro experiment showed that the expression of miR-33a in hepatocytes was significantly inhibited by increasing levels of 25-HC. (**Figs 7 and 8**)

### 5. Analysis of correlations

A significant correlation was found between hepatic expression of miR-33a and plasma level of miR-33a. In vivo, the expression of miR-33a in liver positively correlated with genes involved in cholesterol synthesis (*hmgcr*, *hmgcs*, *cyp51*, *dhcr7* and *srebp-2*). The plasma level of miR-33a was also positively correlated with *cyp51* and *srebp-2* but correlations with the other cholesterol synthetic genes (*hmgcs*, *hmgcr* and *dhcr7*) were not confirmed. In vitro, the expression of miR-33a in hepatocytes showed significant positive correlation with the expression of *hmgcr*, *cyp51*, *dhcr7* and *srebp-2*. (**Table 4**)

**Table 4:** Correlation of miR-33a with cholesterol metabolism parameters.

Pearson value			Cholesterol synthesis					Cholesterol elimination						Lipogenesis			miR-33a ^1^
			*hmgcs*	*hmgcr*	*cyp51*	*dhcr7*	*srebp-2*	*cyp7a1*	*ugt1a3*	*abcg5*	*abcg8*	*abca1*	*lxrα*	*fas*	*srebp-1c*	*gck*	
In vivo	n = 12	L-miR-33a	0.6013*	0.6444*	0.7704**	0.7574**	0.6651*	0.7087**	0.3671	0.6499*	0.3382	-0.2204	-0.0462	0.8423**	0.8151**	-0.6648*	0.9135**
	n = 11	P-miR-33a	0.5840	0.5525	0.6977*	0.5917	0.6112*	0.714*	0.2973	0.6591*	0.9265**	-0.3118	-0.1244	0.7803**	0.8895**	-0.5607	
In vitro	n = 15	C-miR-33a	0.4474	0.554*	0.5626*	0.5318*	0.5225*	0.5899*	0.6439**	\	0.2184	0.5693*	-0.1382	-0.5993*	0.5767*	-0.2340	/

*P<0.05 is statistically significant with one star.

**P<0.01 is statistically very significant with two stars.

^1^ miR-33a: Correlation between liver miR-33a (L-miR-33a) and plasma miR-33a (P-miR-33a) in vivo.

Pearson correlation coefficient larger than zero is positive correlation, while Pearson correlation coefficient less than zero is negative correlation.

With regard to cholesterol elimination, the expression of miR-33a in liver positively correlated with the hepatic expression of *cyp7a1* and *abcg5* but not with the expression of *ugt1a3*, *abcg8*, *abca1* and *lxrα*. Similarly, the abundance of miR-33a in plasma positively correlated with the hepatic expression of *cyp7a1* and *abcg5* as well as *abcg8*. In vitro, the expression of miR-33a in hepatocytes also positively correlated with many genes involved in cholesterol elimination, including *cyp7a1*, *ugt1a3* and *abca1*. (**Table 4**)

Regarding lipogenesis, in vivo, miR-33a levels in liver and plasma positively correlated with the expression of both *fas* and *srebp-1c*. A negative correlation was also found between miR-33a and *gck* gene expression in liver. In vitro, the correlation was different from in vivo findings, with miR-33a in hepatocytes showing a negative correlation with *fas* but positive correlation with *srebp-1c*. (**Table 4**)

## Discussion

Using both in vivo and in vitro experiments, the present study investigated the underlying mechanisms involved in the regulation of cholesterol metabolism in rainbow trout. The transcriptional factor SREBP-2 and the posttranscriptional factor miR-33a were found to be affected in vivo and in vitro, strongly supporting their involvement in cholesterol homeostasis in rainbow trout.

The growth performance was significantly decreased in trout fed plant-based diet, as previously demonstrated in other studies [58,59]. A variety of vegetable-specific compounds [60] and the deficiency in n-3 long-chain polyunsaturated fatty acids (LC-PUFA) and cholesterol in plant-based diet may exert the detrimental effect on growth performance. As expected, the cholesterol level in plasma was decreased in the trout fed plant-based diet, which may be attributed to the decreased supply of cholesterol [61], the soybean protein inclusion [62], or other vegetable substances that exert lowering effect on plasma cholesterol, such as isoflavones [63], phytate [64,65] and phytosterols [66].

In trout fed the plant-based diet, expression of genes involved in cholesterol synthesis (*hmgcr*, *hmgcs*, *cyp51*, *dhcr7*) as well as their master regulator *srebp-2* increased, suggesting a promotion of the synthesis of cholesterol regulated by SREBP-2 to keep cholesterol homeostasis when trout were fed plant-based diet devoid of cholesterol. This concordant upregulation of cholesterol synthetic genes and *srebp-2* was also found in other studies in trout [59] and Atlantic salmon [5,14,15] fed plant-based diet. In the present study, consistent results linking SREBP2 and cholesterol synthesis were also obtained in vitro with the concomitant decreased expression of *srebp-2* and genes involved in cholesterol synthesis in response to increasing levels of 25-HC. 25-HC is an oxysterol produced endogenously during cholesterol hydroxylation. It is often used in cell culture as a reflection of short-term elevation of cellular cholesterol level [67,68]. Both cholesterol and 25-HC could inhibit the activation of SREBP but through different mechanisms [69]. Thus, the graded inhibition of the expression of cholesterol synthetic genes and *srebp-2* by 25-HC indicated that the cholesterol synthesis was finely regulated by the cellular cholesterol content in rainbow trout. This regulation mediated by SREBP-2, which has been well studied in mammals [70] was also conserved in fish. Altogether, these results support the conclusion that cholesterol depletion contributes to enhance the mechanisms of cholesterol synthesis in trout fed plant-based diet.

Unlike cholesterol synthesis, the expression of genes involved in bile acid synthesis and cholesterol excretion (*cyp7a1*, *ugt1a3*, *abcg5*, *abcg8*, *abca1*) were not affected in the present study when trout were fed the plant-based diet. In mammals, it was reported that the expression of *cyp7a1* [31], *abcg5*, *abcg8* [32,71] and *abca1* [72,73] were all subjected to the transcriptional regulation by LXRs. The absence of modulation of the expression of these genes in our in vivo study is therefore in agreement with the expression of *lxrα* that was also not affected by the composition of the diet. A negative impact of plant-based diet on the expression of *cyp7a1*, *abcg8* and *lxrα* had been yet previously recorded in trout fed plant-based diet. However, this regulation was observed after a longer feeding trial, 6-month [59] compared to 10 weeks in the present study, suggesting a progressive and adaptive metabolic response of the fish to the dietary cholesterol deficiency. In accordance with this hypothesis, it has been shown that the expression of *lxr* is not affected by plant-based diet in Atlantic salmon fed during a short period of 10 weeks [5] but significantly influenced after a longer period of two years [74]. In the in vitro experiment, 25-HC failed to increase the expression of *lxrα*. The oxysterols identified as potent natural ligand for LXRs in mammalian cell-based systems are mainly 22(R)-hydroxycholesterol, 24(S),25-epoxycholesterol and 24(S)-hydroxycholesterol [75–77], whereas 25-HC is only a weak activator of LXRs. Therefore, the inactivated expression of *lxrα* by 25-HC in the present study may be attributed to the weak activation of *lxrα* transcription by 25-HC or inadequate condition for *lxrα* gene expression stimulation, in terms of time or stimulus concentration, in the present primary cell culture of hepatocyte experiment. By contrast, the expression of *cyp7a1*, *ugt1a3*, *abcg8* and *abca1* was inhibited by 25-HC, which suggested that other transcriptional or posttranscriptional factors are involved in the regulation of these genes and are not compensated by *lxrα* in the present situation.

Regarding the lipogenic genes, the expression of *fas* was markedly increased in trout fed plant-based diet. This induction of *fas* gene expression by plant-based diet could be one of the reason why plant-based diet usually enhance body lipid content at long term [59]. The impact of vegetable diet on lipid accumulation was unfortunately not confirmed in the present study may be because of the duration of the trial, which was too short to start observing impact on whole body lipid content. However, the expression of *srebp-1c*, which is known as the transcriptional regulator of *fas*, was not affected by plant-based diet in the present study, indicating that other mechanisms may contribute to the regulation of lipogenesis besides SREBP-1c, such as the transcription factors upstream stimulatory factor 1 (USF1) and carbohydrate-responsive element-binding protein (ChREBP) (reviewed by Wang, 2015) [78] or the posttranscriptional regulators miR-122 and miR-370 [79,80].

In addition to its role in the transcriptional regulation of cholesterol catabolism, LXRs were also known to enhance lipogenesis by activating SREBP-1c in mammals [81,82]. Therefore, LXRs were suggested as the sensors of the balance between cholesterol and fatty acid metabolism. In mammals, it has been suggested that 25-HC served as LXR ligand, increasing the expression of *fas* via *srebp-1c* activation [83]. Though the increased expression of *fas* by 25-HC was found in the present study, the expression of *lxrα* and *srebp-1c* was either unaffected or inhibited by 25-HC, suggesting a different mechanism underlying the transcriptional regulation of lipogenesis in fish or at least in primary cell culture of hepatocytes, which merits further investigations in the future. As an important enzyme in the glycolytic pathway, the higher expression of *gck* in trout fed marine diet could be attributed to the higher level of starch, mainly provided by extruded wheat, in the marine diet. Actually, the expression of *gck* in rainbow trout is highly sensitive to the dietary protein to carbohydrate ratio and strongly increase when starch is supplied to the fish [84]. However, the reason why *gck* expression was stimulated in vitro by 25-HC under the concentration of 4mg/L is still elusive and needs further investigations to understand the interlink between cholesterol and glucose metabolism.

In the present study, the expression of miR-33a was consistently modulated as *srebp-2* gene expression both in vivo and in vitro. While increased in trout fed the plant-based diet when no cholesterol is provided to the fish, expression of miR-33a and SREBP-2 decreased in primary cell culture of hepatocytes stimulated with 25-HC. In trout, the miR-33a is located between exons 16 and 17 of the two paralogues encoding SREBP-2 (Position 39884986–39885006 on the NCBI reference NC_035088.1 sequence and position 71698078–71698058 on the NCBI reference the NC_035089.1), confirming the intronic location of miR-33a in the *srebp-2* gene sequence as it is the case in mammals [44]. This suggests that miR-33a may play a conserved role in rainbow trout and mammals, which is synergistically enhancing cellular cholesterol level together with SREBP-2.

In mammals, *abca1* and *cyp7a1* were identified as the direct targets of miR-33a [45,47]. However, neither experiment implemented in the present study shows a consistent regulation between *abca1* and *cyp7a1* gene expression and miR-33a expression. Conversely, positive correlations were even found between *abca1* and miR-33a in vivo and between *cyp7a1* and miR-33a both in vivo and in vitro. Additionally, other genes involved in cholesterol elimination, such as *abcg5* and *ugt1a3*, also showed positive correlations with miR-33a in vivo or in vitro. These results oppose to the assumption that miR-33a directly target several genes involved in cholesterol elimination in mammals. Therefore, further studies are needed to identify the miR-33a-mediated regulation of cholesterol metabolism in rainbow trout in the future.

The potential synergy of miR-33a and SREBP-2 on cholesterol metabolism is supported by the significant statistical correlations found between several cholesterol synthetic genes which are known to be regulated by SREBP-2 and the hepatic abundance of miR-33a, both in vivo and in vitro. These results strengthen the hypothesis that miR-33a may be indirectly involved in the posttranscriptional regulation of cholesterol synthesis in trout. However, as miR-33a is probably co-transcribed with SREBP-2, the correlation between miR-33a and genes involved in cholesterol synthesis might be fortuitous. Therefore, further studies based on the utilization of miR-33a mimic or inhibitor should be conducted to clarify the role of miR-33a in the regulation of the cholesterol metabolism in rainbow trout.

Of note, hepatic and plasma miR-33a level in vivo showed significant positive correlation between each other. Thus, as their hepatic counterpart, circulating miR-33a positively correlates with genes involved in cholesterol synthesis and elimination. Since circulating miRNAs were identified in humans as noninvasive biomarkers of diseases [85–87], for example, cardiac myocyte-associated miR-208b and -499 were highly elevated in plasma from acute myocardial infarction patients [85], the present study suggests that the abundance of miR-33a in plasma could constitute an interesting biomarker of cholesterol metabolism in rainbow trout.

In conclusion, the present study provides new information about the involvement of SREBPs, LXR and miR-33a in the regulation of cholesterol metabolism in fish upon cholesterol supply. SREBP-2 and miR-33a seem to function synergistically to promote cholesterol synthesis in rainbow trout. However, the posttranscriptional regulation of cholesterol catabolism mediated by miR-33a remains questionable in trout, which still needs further study. The transcriptional regulation of cholesterol catabolism by LXR is less susceptible, but other mechanisms may underlie the regulation of cholesterol catabolism in trout. The observation that miR-33a in plasma could be a relevant biomarker of cholesterol metabolism in trout opens promising perspectives of utilization of circulating miRNAs as noninvasive phenotypic biomarkers in aquaculture.

## Supporting information

S1 File Individual raw data and mean ± SD of miRNA and gene expression levels.Raw data for making Fig1,2,5 excel sheet: individual expression of genes involved in cholesterol synthesis, cholesterol elimination and lipogenesis in the liver of rainbow trout fed marine (M) diet and (V) vegetable diet. Fig1 excel sheet: mean and SD of gene expression involved in cholesterol synthesis in liver. Fig2 excel sheet: mean and SD of gene expression involved in cholesterol elimination in liver. Fig5 excel sheet: mean and SD of gene expression involved in lipogenesis in liver. Raw data for making Fig2,4,6 excel sheet: individual expression of genes involved in cholesterol synthesis, cholesterol elimination and lipogenesis in trout hepatocytes stimulated with graded levels of 25-hydroxycholesterol. Fig3 excel sheet: mean and SD of gene expression involved in cholesterol synthesis in trout hepatocytes stimulated with graded levels of 25-hydroxycholesterol. Fig4 excel sheet: mean and SD of gene expression involved in cholesterol elimination in trout hepatocytes stimulated with graded levels of 25-hydroxycholesterol. Fig6 excel sheet: mean and SD of gene expression involved in lipogenesis in trout hepatocytes stimulated with graded levels of 25-hydroxycholesterol. Raw data for making Fig7,8 excel sheet: individual raw data of miR-33a expression in liver and plasma of rainbow trout fed marine (M) diet and (V) vegetable diet and in trout hepatocytes stimulated with graded levels of 25-hydroxycholesterol. Fig7 liver excel sheet: mean and SD of miR-33a expression in liver. Fig7 plasma excel sheet: mean and SD of miR-33a abundance in plasma. Fig8 excel sheet: mean and SD of miR-33a expression in in trout hepatocytes stimulated with graded levels of 25-hydroxycholesterol.
(XLSX)
